# Acupuncture Therapies and Neuroplasticity

**DOI:** 10.1155/2017/6178505

**Published:** 2017-04-27

**Authors:** Cun-Zhi Liu, Jian Kong, KeLun Wang

**Affiliations:** ^1^Acupuncture and Moxibustion Department, Beijing Hospital of Traditional Chinese Medicine Affiliated to Capital Medical University, 23 Meishuguanhou Street, Dongcheng District, Beijing 100010, China; ^2^Beijing Key Laboratory of Acupuncture Neuromodulation, Beijing Hospital of Traditional Chinese Medicine Affiliated to Capital Medical University, 23 Meishuguanhou Street, Dongcheng District, Beijing 100010, China; ^3^Department of Psychiatry, Massachusetts General Hospital (MGH), Harvard Medical School, Charlestown, MA 02129, USA; ^4^SMI, Department of Health Science and Technology, Faculty of Medicine, Aalborg University, Aalborg, Denmark

Neuroplasticity, including dendritic remodeling, synapse turnover, long-term potentiation (LTP), and neurogenesis, is a feature of the brain's response to the environment. This physiological process is engaged in development of brain, skill learning, formation and extinction of memory, and self-repair of neural injuries. Acupuncture has been demonstrated to be effective in many disorders such as stroke, Alzheimer's disease, and pain, the pathologies of which are related to neural plasticity. As a form of peripheral stimulation, acupuncture may relieve the symptoms of patients via mediating on neural plasticity.

This special issue contains 11 manuscripts, of which 3 manuscripts study the mechanism of acupuncture in various pain diseases using animal models. Among these, G.-H. Tian et al. found that electroacupuncture (EA) treatment exerts abirritative effects by inhibiting brain neuronal apoptosis and aberrant astrocyte activation. J.-Y. Wang et al. suggested that EA reduced the effects of the noxious stimulus on pain-related neurons in chronic constrictive injury rats. X.-M. Shao et al. demonstrated that EA can alleviate retrieval of pain memory due to the partial inhibition of cAMP/PKA/CREB signaling pathway. And not only that, but also H. Jiang et al. found that acupuncture could ameliorate depressive-like behaviors by regulating the PKA/CREB signaling pathway in the hippocampus.

In the special issue, there are 4 manuscripts about the neuroprotection of acupuncture on neurologic disease. One manuscript found that musical electroacupuncture therapy performed better than EA treatment in decreasing amyloid-beta levels in the frontal lobe of SAMP8 mice with Alzheimer's disease. The other three explored the molecular mechanisms of acupuncture. Y. Mo et al. suggested EA can greatly promote neuronal function recovery after spinal cord injuries in rats, which may result from upregulating the expression of neurotrophin-3. W. Liu et al. detected that miR-134-mediated LIMK 1 function was involved in EA-induced the hippocampal synaptic plasticity, which served as a contributor to improving spatial reference learning and memory during the recovery stage of ischemic stroke. And H.-Q. Li et al. suggested that EA can improve neurological deficit scores and reduce blood-brain barrier permeability after intracerebral haemorrhage, and the mechanism possibly targets caveolin-1/matrix metalloproteinase/blood-brain barrier permeability pathway.

In addition, there are 2 manuscripts using functional magnetic resonance imaging to explore the mechanism underlying acupuncture treatment. One manuscript investigated how causal influences between brain regions during the rubber hand illusion are modulated by tactile and visual stimuli. The other one investigated neuroplasticity changes induced by a single session of acupuncture therapy in healthy adults, regarding the excitability change on bilateral primary motor cortex and interhemispheric inhibition. Furthermore, J. W. Yang et al. investigated the effect and underlying mechanism of acupuncture on renal sympathetic activity in spontaneously hypertensive rats.

In summary, this issue provides varies evidences presented by diverse authors covering several topics related to advances in acupuncture for mediating neural plasticity. Neural plasticity could be a bridge between acupuncture and various neurological diseases. More in-depth researches are required to reveal the underlying mechanism of acupuncture.

*Cun-Zhi Liu* *Cun-Zhi Liu* *Jian Kong* *Jian Kong* *KeLun Wang* *KeLun Wang*

